# Shiga Toxin Selectively Upregulates Expression of Syndecan-4 and Adhesion Molecule ICAM-1 in Human Glomerular Microvascular Endothelium

**DOI:** 10.3390/toxins12070435

**Published:** 2020-07-03

**Authors:** Elena B. Volokhina, Wouter J. C. Feitz, Lonneke M. Elders, Thea J. A. M. van der Velden, Nicole C. A. J. van de Kar, Lambertus P. W. J. van den Heuvel

**Affiliations:** 1 Department of Pediatric Nephrology, Amalia Children’s Hospital, Radboud Institute for Molecular Life Sciences, Radboud University Medical Center, 6525 GA Nijmegen, The Netherlands; Wouter.Feitz@radboudumc.nl (W.J.C.F.); lonneke.elders@gmail.com (L.M.E.); Thea.vanderVelden@radboudumc.nl (T.J.A.M.v.d.V.); Nicole.vandeKar@radboudumc.nl (N.C.A.J.v.d.K.); Bert.vandenHeuvel@radboudumc.nl (L.P.W.J.v.d.H.); 2 Department of Laboratory Medicine, Radboud University Medical Center, 6525 GA Nijmegen, The Netherlands; 3 Department of Pediatrics, University Hospitals Leuven, 3000 Leuven, Belgium

**Keywords:** hemolytic uremic syndrome, human glomerular microvascular endothelium, Shiga toxin 1, gene expression, heparan sulfate proteoglycans, syndecan-4, ICAM-1

## Abstract

Hemolytic uremic syndrome (HUS) is a severe renal disease that is often preceded by infection with Shiga toxin (Stx)-producing *Escherichia coli* (STEC). The exact mechanism of Stx-mediated inflammation on human glomerular microvascular endothelial cells (HGMVECs) during HUS is still not well understood. In this study, we investigated the effect of Stx1 on the gene expression of proteins involved in leucocyte-mediated and complement-mediated inflammation. Our results showed that Stx1 enhances the mRNA and protein expression of heparan sulfate proteoglycan (HSPG) syndecan-4 in HGMVECs pre-stimulated with tumor necrosis factor α (TNFα). CD44 was upregulated on mRNA but not on protein level; no effect on the mRNA expression of other tested HSPGs glypican-1 and betaglycan was observed. Furthermore, Stx1 upregulated the mRNA, cell surface expression, and supernatant levels of the intercellular adhesion molecule-1 (ICAM-1) in HGMVECs. Interestingly, no effect on the protein levels of alternative pathway (AP) components was observed, although C3 mRNA was upregulated. All observed effects were much stronger in HGMVECs than in human umbilical endothelial cells (HUVECs), a common model cell type used in endothelial studies. Our results provide new insights into the role of Stx1 in the pathogenesis of HUS. Possibilities to target the overexpression of syndecan-4 and ICAM-1 for STEC-HUS therapy should be investigated in future studies.

## 1. Introduction

Hemolytic uremic syndrome (HUS) is a severe renal illness that is characterized by hemolytic anemia, thrombocytopenia, and acute renal failure [1,2]. In most cases, HUS affects children and is preceded by an infection with Shiga toxin-producing *Escherichia coli* (STEC—STEC-HUS) [3]. HUS-causing STEC strains produce type 1 (Stx1) and/or type 2 (Stx2) Shiga toxins [4]. The toxins pass the intestinal wall and enter circulation. They are mainly recognized by the glycolipid receptor globotriaosylceramide (Gb_3_) on target cells [5,6], such as human umbilical vein endothelial cells (HUVECs), human glomerular microvascular endothelial cells (HGMVECs), and human brain microvascular endothelial cells, leading to toxin internalization. The expression of the Gb3 receptor and sensitivity of the endothelium to Stx is increased under pro-inflammatory conditions. To model such conditions in vitro, endothelial cells are pre-incubated with inflammatory mediators, such as tumor necrosis factor α (TNFα) [7].

Shiga toxin has been implicated in protein synthesis inhibition, the induction of signaling pathways, and apoptosis [8,9]. Nevertheless, the mechanisms involved in glomerular microvascular endothelial inflammation, which causes renal damage in HUS, are still not well understood.

In the past, we reported increased numbers of mononuclear and polymorphonuclear leukocytes in the glomeruli of STEC-HUS patients, and another study showed the presence of polymorphonuclear cells in the glomeruli of patients that died during an acute STEC-HUS episode [10,11,12]. A high leucocyte blood count in the acute phase of the disease is related to a prognosis of a worse outcome [13]. Furthermore, both Stx1 and Stx2 induce leucocyte adhesion to the surface of primary HGMVECs, a cell type damaged in HUS. This process is partly abolished by heparinase treatment, indicating an important role for heparan sulfate proteoglycans (HSPGs) in Stx-mediated inflammation [14]. HSPGs consist of core proteins with covalently attached heparan sulfate side chains. The HSPGs are located in the basement membrane and at the cells surface. These molecules play an important role in inflammation, as they are able to bind chemokines and mediate leukocyte rolling, adhesion, and transmigration [15].

Next to cell-mediated inflammation, the role of the complement system in STEC-HUS has been studied. Alternative pathway (AP) complement activation biomarkers have been found in the circulation of children with HUS [16,17]. In in vitro experiments, Stx2 has been shown to bind complement factor H (CFH), an important endogenous AP inhibitor, and, as such, to promote AP activation [18]. Furthermore, Stx1 induced the surface expression of P-selectin and C3 binding to human microvascular endothelium of dermal origin [19].

Thus far, only supportive therapy is available for STEC-HUS. The complement component C5 inhibitor eculizumab, although highly effective in atypical HUS (not preceded with STEC infection) has shown inconsistent results in STEC-HUS patients [20,21]. Therefore, other mechanisms besides C5 activation within and/or outside of complement are likely to be important for STEC-HUS progression, and more research is needed to identify future therapy targets.

In this study, we hypothesized that Stx is able to alter the gene expression of HSPGs and/or AP complement genes in endothelial cells and, in this way, promote inflammation and endothelial damage seen in HUS. To investigate this, primary HGMVECs from different donors were used and compared with HUVECs. As the endothelium of the kidney is mainly involved in the pathogenesis of HUS, differences between these endothelial cell types could be of high importance for the understanding of the disease, and, to date, not many studies have been done on Stx with primary HGMVECs. There are numerous different HSPGs expressed in human tissues, but here, we focused on the HSPGs CD44, syndecan-4, glypican-1, and betaglycan because these are known to be expressed on the glomerular endothelial surface and thus may be relevant in HUS [15]. We also analyzed the gene expression of intercellular adhesion molecule-1 (ICAM-1), a major leucocyte adhesion molecule, and the alternative pathway complement components C3, CFH, complement factor I (CFI) and membrane cofactor protein (MCP)/CD46, as altered expression of these genes may lead to local inflammation-mediated damage in glomeruli.

## 2. Results

### 2.1. Stx1 Upregulates Syndecan-4 mRNA and Protein Expression in HGMVECs

**Figure 1:**
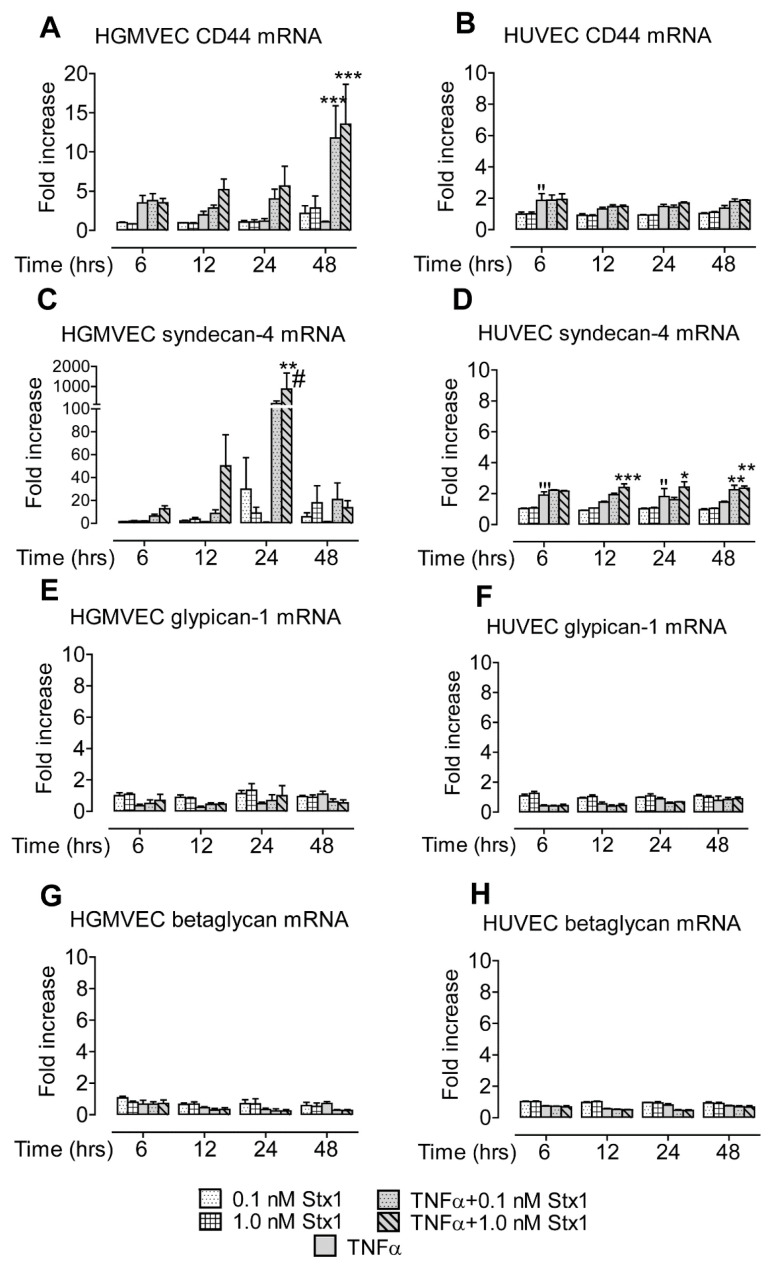
Effect of type 1 Shiga toxin (Stx1) on the mRNA expression of different heparan sulfate proteoglycans (HSPGs) in human glomerular microvascular endothelial cells (HGMVECs) and human umbilical endothelial cells (HUVECs). (**A**,**C**,**E**,**G**) Primary HGMVECs and (**B**,**D**,**F**,**H**) HUVECs from three different donors were incubated with 0.0, 0.1, or 1.0 nM of Stx1 with or without 24 h pre-stimulation with 10 ng/mL of tumor necrosis factor α (TNFα). Mean values and SE are given. Statistically significant differences relative to the untreated control (**‘**), the sample treated with TNFα alone (*), or the sample of the same condition but a different Stx1 concentration (#) are indicated with single (*p* < 0.05), double (*p* < 0.01), or triple (*p* < 0.001) characters, respectively.

The effect of Stx1 on mRNA levels of CD44, syndecan-4, glypican-1, and betaglycan on primary HGMVECs and HUVECs from three different donors was analyzed. No changes in HSPG mRNA expression were observed when HGMVECs or HUVECs were stimulated with Stx1 alone (Figure 1). When cells were exposed to 10 ng/mL of TNFα for 24 h without Stx1 stimulation, the mRNA expression of all HSPG genes was not significantly affected in HGMVECs (Figure 1A,C,E,G), while only the expression of CD44 and syndecan-4 was marginally increased in HUVECs (<2-fold at all time points; Figure 1B,D,F,H). The stimulation of HGMVECs with Stx1 after exposure to TNFα resulted in the profound mRNA upregulation of CD44 (up to 13.5-fold at 48 h) and the even stronger upregulation of syndecan-4 (up to 869-fold at 24 h) (Figure 1A,C). When HUVECs were used, a minor upregulation of syndecan-4 mRNA was observed (maximum of 2.4-fold at 24 h) (Figure 1D).

**Figure 2:**
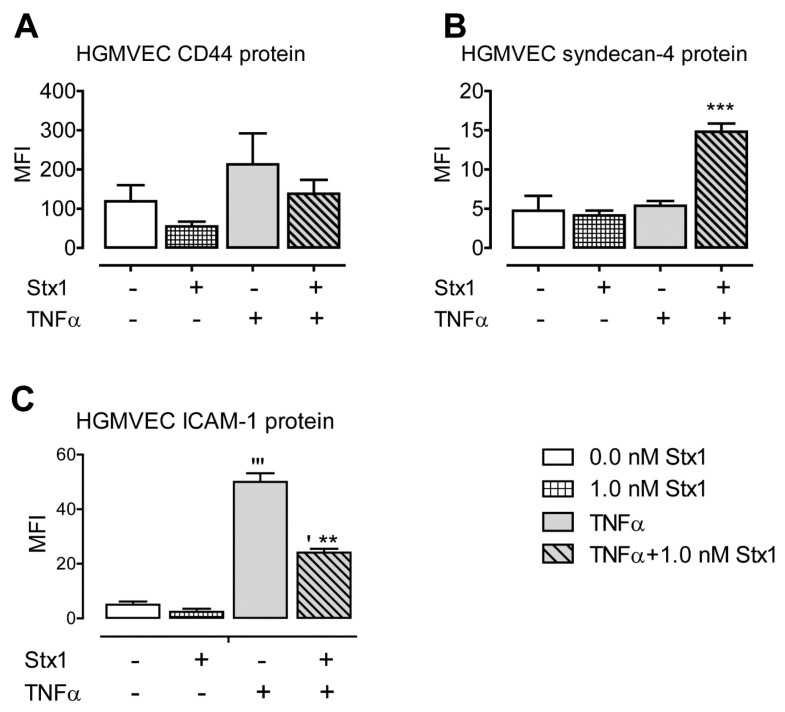
Effect of Stx1 on cell surface expression of (**A**) CD44, (**B**) syndecan-4, and (**C**) intercellular adhesion molecule-1 (ICAM-1) proteins in HGMVECs. Primary HGMVECs from four different donors (CD44 and syndecan-4) and two donors (ICAM-1) were incubated with 1.0 nM Stx1 for 24 h with or without 24 h pre-stimulation with 10 ng/mL of TNFα. Mean values and SE are given. Statistically significant differences relative to the untreated control (**‘**) or the sample treated with TNFα alone (*) are indicated with single (*p* < 0.05), double (*p* < 0.01), or triple (*p* < 0.001) characters, respectively.

Next, we analyzed whether the mRNA upregulation of CD44 and syndecan-4 in HGMVECs was translated into a higher expression of these HSPGs on the cell surface. Incubation times of 24 and 48 h were chosen, as mRNA upregulation was observed to be highest in these conditions. The incubation of cells for 48 h with TNFα and 1.0 nM Stx did not yield enough viable cells for reliable fluorescence-activated cell sorting (FACS) analysis, so only 24 h of incubation were analyzed. No significant upregulation compared to untreated samples was observed when cells were treated with Stx1 alone. When HGMVECs were stimulated with Stx1 after TNFα exposure, only syndecan-4 protein expression was upregulated (>3-fold; Figure 2A,B).

Altogether, we demonstrated that Stx1 strongly upregulates the mRNA expression of the HSPG proteins CD44 and syndecan-4 in HGMVECs after TNFα exposure. A much lower effect and only for syndecan-4 was seen when HUVECs were used. The mRNA levels of glypican-1 and betaglycan were unaffected in both cell types. At the protein level, only syndecan-4 upregulation on the surface of HGMVECs was observed.

### 2.2. Stx1 Increases ICAM-1 mRNA Expression and Secretion in Primary HGMVECs

**Figure 3:**
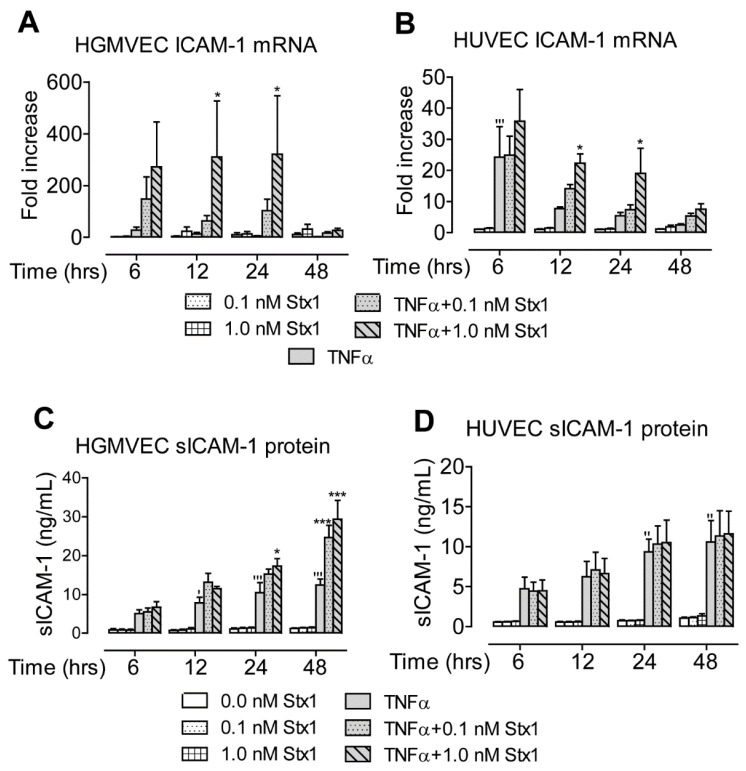
Effect of Stx1 on ICAM-1 mRNA expression and soluble ICAM-1 (sICAM-1) protein levels in the supernatant of (**A**,**C**) HGMVECs and (**B**,**D**) HUVECs. Primary HGMVECs and HUVECs from three different donors were incubated with 0.0, 0.1, and 1.0 nM of Stx1 with or without 24 h pre-stimulation with 10 ng/mL of TNFα. Mean values and SE are given. Statistically significant differences relative to the untreated control (**‘**) or the sample treated with TNFα alone (*) are indicated with single (*p* < 0.05), double (*p* < 0.01) or triple (*p* < 0.001) characters, respectively.

Next, we analyzed whether Stx1 affects the expression of ICAM-1, a major leucocyte recruitment and adhesion molecule. At the mRNA level, no effect was observed when HGMVECs or HUVECs were incubated with Stx1 alone (Figure 3A,B). Exposure to 10 ng/mL of TNFα without Stx1 stimulation for 6 h led to a significant increase of ICAM-1 mRNA levels in HUVECs (24-fold), though this effect diminished after longer incubation times (Figure 3B). The stimulation of cells with Stx1 after TNFα yielded an increase in ICAM-1 mRNA in both HGMVECs (maximum of 320-fold) and HUVECs (maximum of 22-fold) at 12 and 24 h of incubation (Figure 3A,B). The ICAM-1 mRNA levels decreased after 48 h of incubation in both cell types.

ICAM-1 protein expression was not increased on the surface of HGMVECs after Stx1 incubation alone (Figure 2C). However, exposure to TNFα alone led to a 10-fold upregulation of ICAM-1 protein levels on the surface. Stimulation with Stx1 after TNFα yielded lower level than TNFα alone, though it was still five times higher compared to unstimulated control cells (Figure 2C).

Because soluble ICAM-1 (sICAM-1) is known to play an important role in inflammation, we also measured concentrations of sICAM-1 protein in the culture supernatant. This was not affected by stimulation with Stx1 alone in HGMVECs and HUVECs (Figure 3C,D). Exposure to TNFα alone led to an increase (maximum of 12-fold) in protein levels after 48 h of incubation in both cell types. The incubation of HGMVECs with Stx1 after TNFα led to an increase (maximum of 29-fold) in the culture supernatant after 48 h of incubation (Figure 3C), while no effect was seen in HUVECs (Figure 3D).

Altogether, the exposure of cells to TNFα in combination with treatment with Stx1 caused the upregulation of ICAM-1 mRNA levels. This translated into higher sICAM-1 levels in the supernatant when HGMVECs were used.

### 2.3. Stx1 Increases mRNA, but Not Protein Expression of Complement Component C3

The dysregulation of the AP of the complement system and as result inflammation and damage to the endothelium has been suggested as a possible mechanism for the pathogenesis of STEC-HUS. Next, we analyzed the effect of Stx1 on the expression of the AP components C3, CFI, CFH, and MCP/CD46.

**Figure 4:**
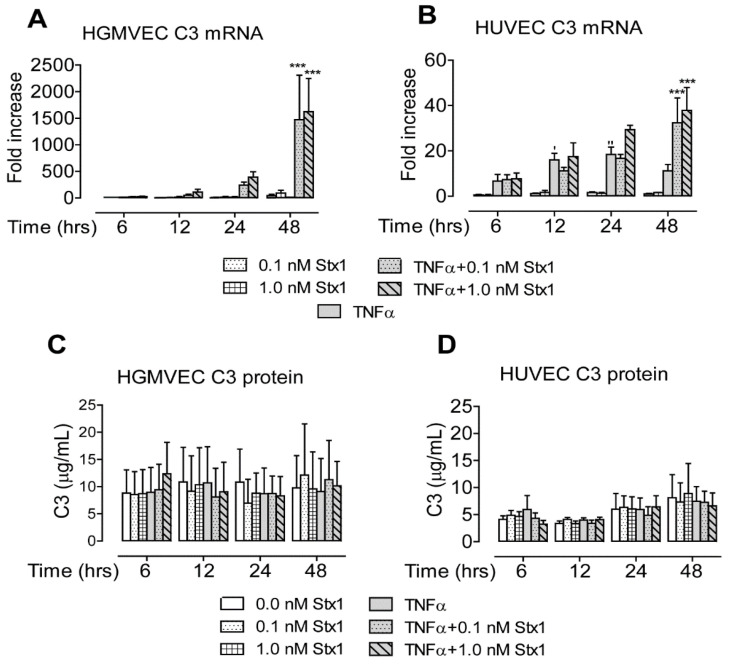
Effect of Stx1 on complement C3 mRNA and protein expression in (**A**,**C**) HGMVECs and (**B**,**D**) HUVECs. Primary HGMVECs and HUVECs from three different donors were incubated with 0.0, 0.1, and 1.0 nM of Stx1 with or without 24 h pre-stimulation with 10 ng/mL of TNFα. Mean values and SE are given. Statistically significant differences relative to the untreated control (**‘**) or the sample treated with TNFα alone (*) are indicated with single (*p* < 0.05), double (*p* < 0.01) or triple (*p* < 0.001) characters, respectively.

No effect on C3 was observed with Stx1 incubation alone in HGMVECs or HUVECs (Figure 4A,B). Exposure to 10 ng/mL of TNFα alone led to the upregulation of C3 mRNA expression in HUVECs (maximum of 19-fold at 24 h) (Figure 4B). The incubation of cells with Stx1 after exposure to TNFα led to a profound increase in C3 mRNA levels after 48 h in HGMVECs (maximum of 1623-fold) and HUVECs (maximum of 37-fold) (Figure 4A,B). No effect on the mRNA expression of CFH, CFI, and MCP/CD46 in HGMVECs or HUVECs was observed (data not shown).

When C3 protein concentrations were measured in the culture supernatant, no effect of Stx1 stimulation with or without TNFα was observed in HGMVECs or HUVECs (Figure 4C,D).

Altogether, of the measured complement components, only C3 was upregulated at the mRNA level by Stx1 in both cell types. No increase of C3 concentration in the culture supernatant was observed.

## 3. Discussion

In the present communication, we report that the stimulation of HGMVECs with Stx1 after TNFα exposure leads to the increased expression of syndecan-4 on the cell surface and of ICAM-1 in a culture supernatant. Interestingly, under the same conditions, CD44 and complement component C3 demonstrated increased levels of mRNA expression that were not translated into higher protein levels. For both, CD44 and C3 mRNA levels were highest at 48 h of incubation (Figure 1A and Figure 4A), while mRNA levels of syndecan-4 and ICAM-1 were already high at 24 h incubation (Figure 1C and Figure 3A). In addition to the upregulation of gene expression, Stx1 also induces ribosomal injury and protein synthesis inhibition by cleaving a specific adenine nucleobase from the 28S RNA of the 60S subunit of the ribosome [22,23,24]. Therefore, a longer incubation period necessary for increased CD44 and C3 mRNA production could give more time for ribosomal injury to accumulate. High CD44 and C3 mRNA levels may therefore not be efficiently translated into high protein levels by inactivated ribosomes. It should be mentioned that we used a 1.0 nM concentration of Stx1 for 24 and 48 h to study HSPG protein expression levels on the cell surface. Incubation with 0.1 nM for 48 h may have resulted in less ribosomal injury while still allowing for sufficient mRNA and protein production to see CD44 protein upregulation on the cells. Interestingly, in the case of C3, which gives a similar mRNA upregulation pattern as CD44 (with the maximum mRNA measured at 48 h), no increase of C3 protein in the medium at 0.1 nM was found at 48 h of incubation.

Stx was shown to enhance the mRNA stability of some transcripts, e.g., of vasoconstrictor endothelin-1 in bovine aortic endothelial cells. This may explain how strong mRNA upregulation may occur despite protein synthesis inhibition, as seen for CD44, syndecan-4, ICAM-1, and C3. Furthermore, in human microvascular endothelial cells of dermal origin, Stx increased both mRNA stability and association with the ribosomes of chemokine receptor type 4 (reviewed by Petruziello-Pellegrini et al.) [25]. This enhanced affinity to ribosomes may have facilitated high protein upregulation for syndecan-4 and ICAM-1. The exact underlying mechanism of the selective effect of Stx1 on the expression of the genes studied here should be investigated in the future. In this study, different proteoglycans were investigated, and the results showed different effects of Stx1 on gene expression dependent on the HSPGs studied. CD44 and syndecan-4 showed mRNA upregulation, while betaglycan and glycipan-1 showed no significant effect after Stx1 and TNFα. Of all the studied HSPGs, only syndecan-4 was upregulated on both mRNA and protein levels. Previously, we showed a role of heparan sulphates in Stx-mediated leucocyte recruitment, and it is likely that endothelial HSPGs interact with L-selectin on the surface of neutrophils [26]. The upregulation of the HSPG syndecan-4 by Stx1 may therefore enhance neutrophil rolling and, in this way, contribute to inflammation. Next to leucocyte recruitment, syndecan-4 is involved in mitogen-activated protein kinase (MAPK) signaling. Furthermore, it is a receptor for vascular endothelial growth factors (VEGF) and platelet-derived growth factors, and it is one of the proteins connecting extracellular matrix and cytoskeletal signaling proteins [27]. A possible role of these syndecan-4 functions in STEC-HUS pathogenesis should be investigated in the future. Moreover, the ectodomain of syndecan-4 is continuously cleaved and released from the cell surface, a process which is intensified under inflammatory conditions. Interestingly, this ectodomain can be cleaved into fragments by thrombin. These fragments have been shown to alter the endothelial barrier function in the lungs [28]. Microthrombi formation and thrombin release is a hallmark of HUS. The enhanced formation of syndecan-4 fragments could thus contribute to endothelial damage after STEC infection.

The upregulation of syndecan-4 in pro-inflammatory conditions has been described before. When HUVECs were incubated with lipopolysaccharides and interleukin-1β (IL-1β), syndecan-4 mRNA was increased, while syndecan-2 expression decreased and syndecan-1 and -3 were unaffected [29]. Here, we showed the upregulation of syndecan-4 under the influence of Stx1 in HGMVECs for the first time.

Next, we saw the high upregulation of ICAM-1. On the cell surface, ICAM-1 expression was increased when cells are treated with TNFα and Stx1. These findings are in line with previously published data showing that the protein expression of ICAM-1 and VCAM-1 is upregulated by Stx1 in HUVECs in vitro after TNFα pre-stimulation [30]. Interestingly, the surface expression of both ICAM-1 and VCAM-1 decreased on human microvascular intestinal endothelial cells stimulated with 10 ng/mL of Stx1 or Stx2 with and without the combination of 2 ng/mL of TNFα and IL-1β [31]. Thus, the upregulation of these adhesion molecules may depend on the cell type.

ICAM-1 on the endothelial surface interacts with lymphocyte function-associated antigen-1 (LFA-1) and macrophage antigen-1 (Mac-1) on leucocytes and, through this, facilitates leukocyte adhesion and trans-endothelial migration. The upregulation of ICAM-1 on cells is likely to enhance leucocyte-mediated inflammation and its associated damage. Previous studies have shown that ICAM-1 is important for leucocyte recruitment in TNFα-stimulated HUVECs [32]. In this study, we showed, for the first time, that soluble ICAM-1 levels are also increased upon Stx1 stimulation after TNFα. The sICAM-1 is released in response to endothelial damage, and its increased levels are associated with several pathologies including cardiovascular disease, cancer, and auto-immune diseases. Still, the role for sICAM-1 in inflammation is not clear and controversial. It is thought to compete with the cell-bound ICAM-1 for LFA-1 and Mac-1 interactions, and, via this way, inhibit leucocyte recruitment. Nevertheless, sICAM-1 has been reported to induce cytokine production, including that of TNFα and macrophage inflammatory protein-2, and NF-κB activation in several cell types [33].

Up until now, STEC-HUS treatment has remained symptomatic, with 5–10% of patients losing renal function in time and a mortality of 2–5% in the acute phase of the disease. Complement inhibition therapy (eculizumab), approved for the less frequent but more severe atypical HUS, has been considered [34,35]. Even though eculizumab is highly effective in atypical HUS, it is not approved in patients with STEC-HUS [20,21]. Interestingly, we did not find the upregulation of complement proteins in response to Stx1. Our data on ICAM-1 and HSPGs in combination with the data published by others indicate that cell-mediated inflammation may be of more importance in the development of STEC-HUS than complement-mediated inflammation.

The targeting of syndecan-4 and L-selectin could be a promising option for the inhibition of leucocyte-mediated and complement-mediated inflammation in the future. The therapeutic inhibition of selectins has shown promising results in a porcine model of renal ischemia reperfusion injury including decrease in cellular inflammation [36]. If successful in humans, timely selectin inhibition could prevent or limit cell-mediated damage in HUS. Not only could it counteract HSPG-mediated cell recruitment enhanced by Stx by blocking L-selectin, it could also diminish complement damage via C3 binding to P-selectin, as published previously [19]. Extensive studies have been performed to target ICAM-1/LFA-1 interactions, which may also be a promising approach to target cell inflammation in STEC-HUS in the future [37].

Altogether, this preliminary work presents observations that give new insight in the pathogenesis of STEC via the alteration of expression of proteins involved in glomerular endothelial inflammation. However, the underlying mechanisms behind our observations have to be addressed and investigated in the future.

## 4. Materials and Methods

### 4.1. Cell Culture

The experiments were performed using human endothelial cells obtained from adult renal tissue that were removed due to medical reasons or isolated from human umbilical veins. The described studies were approved by the appropriate ethics committee and were therefore performed in accordance with the ethical standards laid down in the appropriate version of the 1964 Declaration of Helsinki. All donors gave their informed consent prior to inclusion in this study.

Primary HGMVECs were obtained from human kidneys of three donors and cultured as described previously [38]; passages 8–10 were used for experiments. HUVECs from three donors were harvested according to a previously described method [39], and passages 3–4 were used for experiments. Confluent cells were stimulated for 6, 12, 24, and 48 h with Stx1 (0.0, 0.1, and 1.0 nM) with or without 24 h preincubation with 10 ng/mL of TNFα (Roche Diagnostics). Stx1 was a kind gift from M. Bielaszewska (University of Münster, Münster, Germany) and was endotoxin-free, as determined by a Limulus assay.

### 4.2. RNA Isolation and cDNA Synthesis

To isolate total RNA, approximately 1 × 10^6^ endothelial cells were resuspended in 1 mL of Trizol (Invitrogen, Carlsbad, CA, USA). After the addition of 200 μL of chloroform and centrifugation (15 min, 15,700× *g*, 4 °C), the upper aqueous phase was mixed 1:1 with isopropanol. RNA pellets were collected by centrifugation (15 min, 15,700× *g*, 4 °C) and washed with 0.5 mL of cold 70% ethanol; after centrifugation (5 min 7500× *g*, 4 °C), ethanol was removed with the use of a flame-drown Pasteur pipette. The pellet was air-dried for 30 min, resuspended in 50 μL of RNAse-free water, and incubated for 10 min at 65 °C. RNA preparations were stored at −80 °C. To generate cDNA, 1 μg of RNA was reverse transcribed in a 20 μL reaction mix that contained 0.5 μg of random primers, 0.5 μg Oligo dT, 20 U RNAsin (all purchased from Promega, Madison, WI, USA), and 0.25 mM dNTPs, 10 mM DTT, and 200 U M-MLV reverse transcriptase and First-Strand Buffer (all from Invitrogen). The cDNA synthesis program consisted of the following steps: 10 min at 20 °C, 45 min at 42 °C, and 10 min at 95 °C. cDNA was diluted five times prior to further analysis.

### 4.3. Quantitative Real-Time PCR

TaqMan Gene Expression Assays to analyze syndecan-4 (hs00173409_m1), glypican-1 (hs00164830_m1), CD44 (hs00611256_m1), betaglycan (hs01114253_m1), ICAM-1 (Hs00164932_m1), C3 (hs00163811_m1), CFI (hs00173409_m1), CFH (hs00164830_m1), and MCP (hs00611256_m1) were purchased from Applied Biosystems, Foster City, CA, USA. The porphobilinogen deaminase (PBGD) housekeeping gene was used as a reference, and it was amplified by using forward primer (5′-GGCAATGCGGCTGCAA-3′) and reverse primer (5′-GGGTACCCACGCGAATCAC-3′); the PBGD probe sequence (5′-CTCATCTTTGGGCTGTTTTCTTCCGCC-3′) carried Cy5 reporter fluorophore and BHQ-2 quencher fluorophore. PBGD primers and probes were obtained from Biolegio. The 20 μL of qPCR mix contained 5 μL of cDNA, 1 unit of TaqMan gold, the TaqMan 1000 RXN buffer A (both purchased from Applied Biosystems), 5 mM MgCl_2_, 0.5 mM dNTPs (Invitrogen), 0.3 pM PBGD forward primer, 0.3 pM PBGD reverse primer, 0.3 pM PBGD probe, and 1 μL of the TaqMan Gene Expression Assay. The amplification reaction was performed in 96-well plates (Bio-Rad) in a Bio-Rad qPCR machine. The qPCR reaction consisted of the following steps: 10 min at 95 °C, followed by 40 cycles consisting of 15 s at 95 °C and 1 min at 60 °C. The number of cycles needed for the amplification plot to reach the threshold limit (Ct value) was used for quantification; data analysis was performed with the use of Bio-Rad CFX Manager (Bio-Rad, Hercules, CA, USA) software.

### 4.4. Protein Analyses

The surface expression analysis of HSPGs and ICAM-1 was performed using FACS. The syndecan-4 antibody (sc-9499, Santa Cruz Biotechnology, Dallas, TX, USA) and the CD44 antibody (553131, BD Biosciences, Franklin Lakes, NJ, USA), in combination with donkey-anti-goat (A-110055) and goat-anti-rat (A-1106) Alexa 488 conjugates (both from Molecular Probes, Eugene, OR, USA), were used. ICAM-1 surface expression was analyzed using an ICAM-1 FITC conjugate (F7143, Dako). The protein levels of ICAM-1 in the culture supernatant were measured by a sandwich ELISA using a Human ICAM-1/CD54 DuoSet kit (R&D Systems, Minneapolis, MN, USA). The protein levels of the C3 culture supernatant were determined by sandwich ELISA, using goat anti-C3 polyclonal antiserum (A213, CompTech, Tyler, TX, USA) and horseradish peroxidase (HRP)-conjugated goat antibodies against C3 (55237, MP Biomedicals, Irvine, CA, USA).

### 4.5. Statistical Analysis

Statistical analysis was performed using a two-way ANOVA or one-way ANOVA as appropriate. A *p*-value of 0.05 was set as statistically significant. Data are expressed as mean values +/− standard error (SE).
